# Identification and characterisation of two functional antibiotic MATE efflux pumps in the archaeon *Halorubrum amylolyticum*

**DOI:** 10.1038/s44259-024-00036-5

**Published:** 2024-08-02

**Authors:** Asma A. Fakhoury, Thomas P. Thompson, Khondaker Miraz Rahman, Julianne Megaw, Matthew I. McAteer, Timofey Skvortsov, Stephen A. Kelly, Brendan F. Gilmore

**Affiliations:** 1 Biofilm Research Group, School of Pharmacy, Queen’s University Belfast, Medical Biology Centre, 97 Lisburn Road, Belfast, BT9 7BL UK; 2 Department of Pharmaceutical Chemistry, Faculty of Pharmaceutical Sciences, The Hashemite University, Zarqa, Jordan; 3 Institute of Pharmaceutical Science, King’s College London, 150 Stamford Street, London, SE1 9NH UK; 4 Institute for Global Food Security, School of Biological Sciences, Queen’s University Belfast, 19 Chlorine Gardens, Belfast, BT9 5DL UK

**Keywords:** Archaeal biology, Antimicrobial resistance

## Abstract

Multidrug efflux pumps have been found to play a crucial role in drug resistance in bacteria and eukaryotes. In this study, we investigated the presence of functional multidrug and toxic compound extrusion (MATE) efflux pumps, inferred from whole genome sequencing, in the halophilic archaeon *Halorubrum amylolyticum* CSM52 using Hoechst 33342 dye accumulation and antimicrobial sensitivity tests in the presence and absence of efflux pump inhibitors (EPIs). The whole genome sequence of *H. amylolyticum* CSM52 contained two putative MATE-type efflux pump genes, which may contribute to the inherent resistance to conventional antimicrobial agents reported in archaea. Antimicrobial susceptibility of the wild-type *H. amylolyticum* CSM52 testing revealed a lack of sensitivity to a wide range of antimicrobials, including glycopeptides, aminoglycosides, macrolides, fluoroquinolones, tetracycline, and chloramphenicol. However, the presence of EPIs, such as thioridazine, fluoxetine, and chlorpromazine, significantly increased the susceptibility *of H. amylolyticum* CSM52 to a number of these antimicrobials, indicating the potential involvement of efflux pumps in the observed resistance. A molecular modelling study with EPIs and substrate antimicrobials provided important insights into the molecular interactions with the putative transporter. It suggests that the occupancy of the transporter channel by EPIs has the potential to impact the efflux of antimicrobials. Phylogenetic analysis of the amino acid sequences of both MATE pumps showed low similarity with bacterial representatives, suggesting the presence of novel and distinct MATE efflux pumps in archaea. Our findings provide the first experimental evidence of active antibiotic efflux mechanisms in archaea and their potential roles in antimicrobial resistance, broadening our understanding of mechanisms of archaeal antimicrobial resistance, an overlooked aspect of AMR research.

## Introduction

Multidrug efflux pumps are crucial components of cellular defence mechanisms, responsible for extruding a broad range of substances from toxins to antimicrobials, thereby safeguarding the cells from various toxic compounds. Their role extends beyond defence, since these pumps facilitate the removal of secondary metabolites, waste, and other toxic substances including detergents and heavy metals^1,2^. Multidrug efflux pumps facilitate processes such as adaptation during infections^3^, biofilm formation^4^, and adherence in species like *Acinetobacter baumannii* and *Pseudomonas aeruginosa*^5^.

While bacterial and eukaryotic efflux pumps have been the focus of extensive research, their archaeal counterparts remain relatively under-studied. Bartolucci et al. provided a comprehensive review article on archaeal genomic responses to toxic compounds, highlighting the presence of both primary and secondary multidrug transporters, with a noted abundance in the Euryarchaeota over the Crenarchaeota^6^. Additionally, the SMR transporter in *Halobacterium salinarum*^7^, and the multidrug and toxin extrusion (MATE) type efflux pump in *Pyrococcus furiosus*^8^, have been examined for their potential role in proton coupling and drug resistance^9^. However, studies on the functional characterisations of these transporters are sparse, with few archaeal transporters experimentally demonstrated and no archaea-specific transporter families definitively identified. Previous work by Miyauchi et al. reported the presence of a doxorubicin efflux pump in *Haloferax volcanii* similar to the mammalian P-glycoprotein, which first suggested the existence of such mechanisms in haloarchaea^10^.

Functional drug efflux systems in archaea have implications which extend beyond basic cellular physiology. With emerging evidence that archaea are a critical component of the human microbiota^11,12^, and are potentially involved in diseases such as periodontitis, brain abscess and Parkinson’s disease^13–15^, understanding archaeal efflux mechanisms in the context of antimicrobial resistance becomes increasingly important. This is further emphasised by the increasing recognition of archaea as potential reservoirs of antimicrobial resistance genes^16^. Previous work by Fuchsman et al.^17^ demonstrated the dynamic nature of microbial genetic exchanges, with environmental factors significantly influencing horizontal gene transfer (HGT) between archaea and bacteria^17^. As such, HGT could play a pivotal role in the evolution and functionality of archaeal efflux systems, potentially enhancing their adaptability in diverse habitats. Moreover, recent research has demonstrated that archaeal culture media extracts can induce bacterial virulence factor production through cross-domain signalling^18^, highlighting the intricate interplay between archaea and bacteria in microbial consortia. These emerging trends underscore the need for a deeper understanding of the complex interactions between archaea and other domains of life, recognising their importance in both environmental microbiomes and potential clinical contexts.

In this study, we build upon previous investigations to provide a detailed physiological characterisation of antibiotic resistance in *Halorubrum amylolyticum* CSM52. Our results provide the first experimental demonstration of active antibiotic efflux mechanisms in archaea. This work contributes to current understanding of potential mechanisms of the inherent antimicrobial resistance typically exhibited by archaea, and suggests the existence of distinct, previously undiscovered efflux systems within this domain. To achieve this, we employed whole genome sequencing (WGS) data to identify genes encoding two putative MATE efflux pumps, and using a combination of dye accumulation assays, antimicrobial susceptibility testing, molecular modelling, molecular cloning in *E. coli*, and molecular phylogenetic analysis, confirmed their role in active antibiotic efflux and in the observed inherent resistance of archaea to antibiotics. The significance of these findings extends beyond basic cellular physiology, emphasising the potential influence and consequences of antimicrobial exposure on archaea within both extreme environments and clinical settings, with respect to antimicrobial resistance. It also progresses our understanding of the potential role of archaea as reservoirs of antimicrobial resistance genes in complex microbiota, which is an increasingly pertinent consideration given the evidence of extensive HGT between archaea and bacteria. This study, therefore, provides critical foundational knowledge that may guide future research on the inter-domain interactions that shape microbial communities and the spread of antimicrobial resistance.

## Results

### Genomic sequencing and annotation

**Fig. 1:**
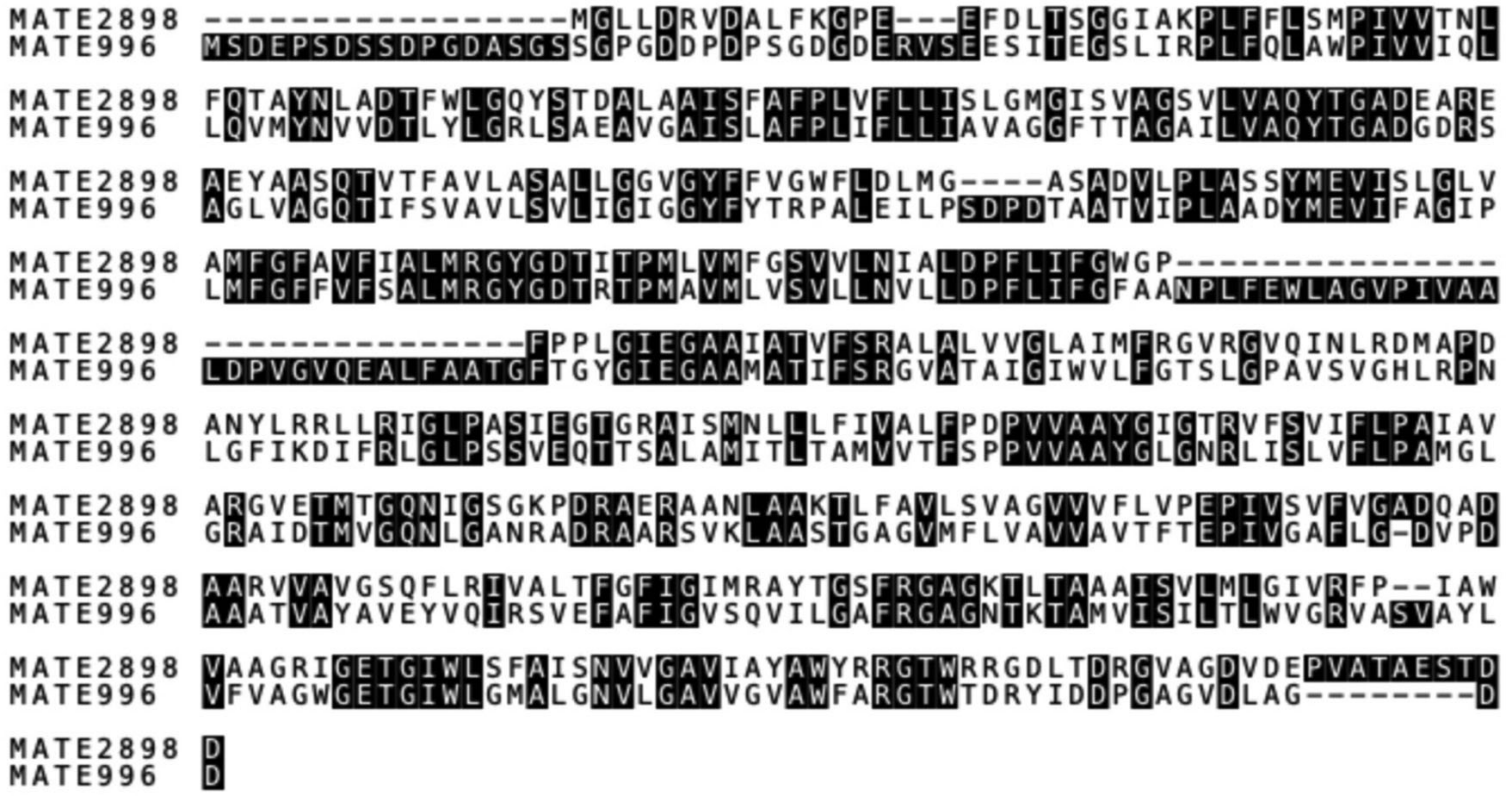
Sequence alignment of MATE-996 and MATE-2898 proteins. The alignment was performed using the ClustalW algorithm and visually represented using Boxshade. Identical residues between the two sequences are highlighted in black, and differences in the sequences are shown in white. The overall sequence identity and similarity provide insights into the evolutionary relationship and potential functional similarities between the two MATE proteins.

The strain *H. amylolyticum* CSM52 was first isolated from a Triassic salt mine in Carrickfergus, Northern Ireland—as described previously^19,20^. Following DNA extraction and high-throughput sequencing using the Illumina MiSeq/NovaSeq platform, the genome was assembled and annotated by uploading to the RAST server (rast.nmpdr.org). This is a fully automated service for annotating bacterial and archaeal genomes and uses subsystem-based assertions to identify and assign functions to protein-encoding genes^21^ the results of which are summarised in Supplementary Table 1. This revealed a diverse array of transporters, predominantly ABC transporters, which facilitate the movement of a wide range of substrates across the cellular membrane. These include transporters for ferric iron, maltose/maltodextrin, ribose, glycerol-3-phosphate, multiple sugars, excinuclease components, nitrate/sulphonate/bicarbonate, phosphate, tungstate, dipeptides, branched-chain amino acids, phosphonates, oligopeptides, methionine, urea, and vitamin B12. Of particular interest, were peg.996 and peg.2898, identified as part of the ‘Multi antimicrobial extrusion protein (Na (+)/drug antiporter), MATE family of multidrug resistance (MDR) efflux pumps. Following an alignment, these two protein sequences were noted to have a 45.17% similarity (Fig. 1).

### Hoechst dye accumulation in *H. amylolyticum* CSM52

**Fig. 2:**
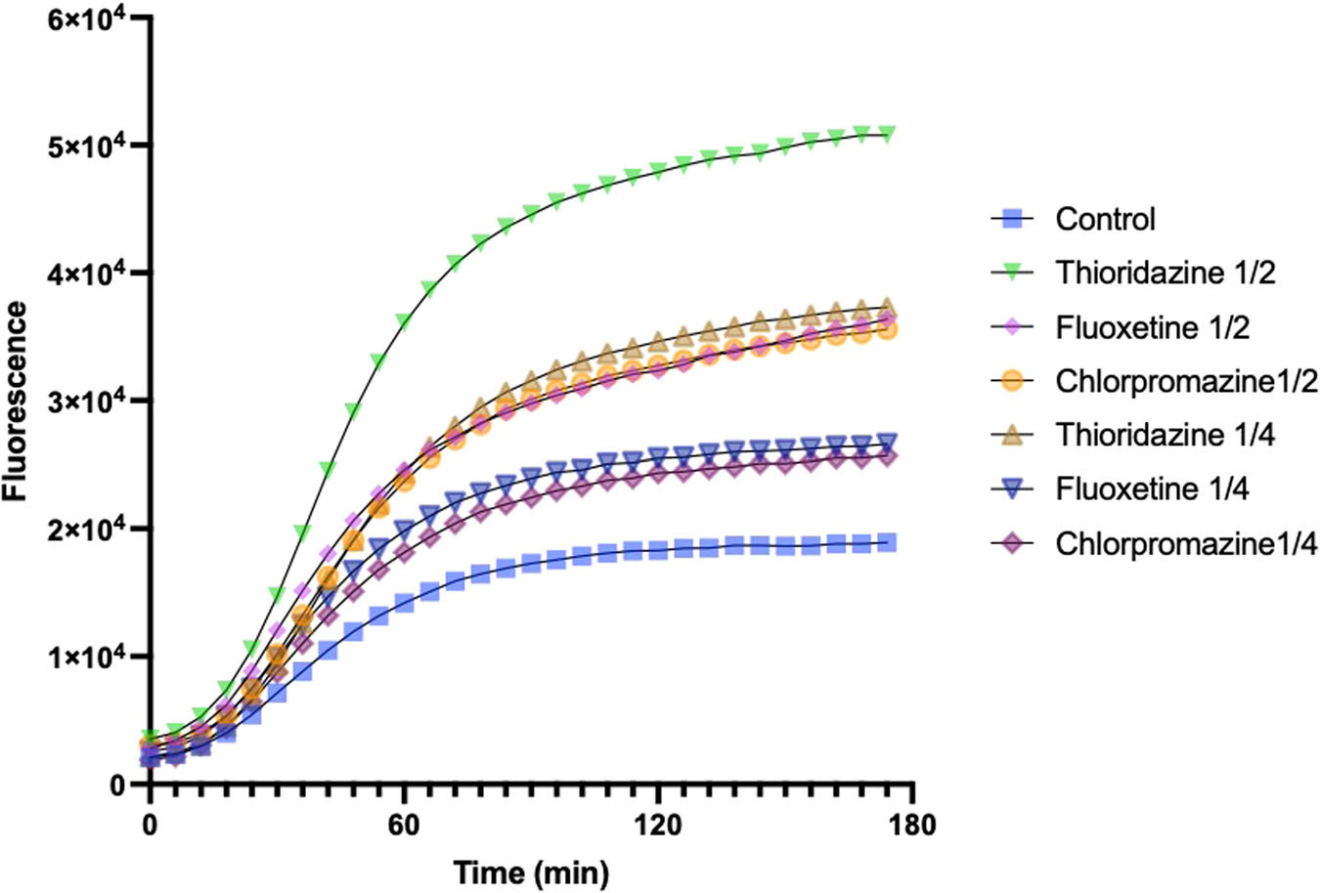
Variation in Hoechst dye accumulation in *H. amylolyticum* CSM52 in the presence of ½ MIC and ¼ MIC of three EPIs thioridazine, chlorpromazine and fluoxetine, over 3 h incubation time. Results are based on eight replicates (*n* = *8*).

**Fig. 3:**
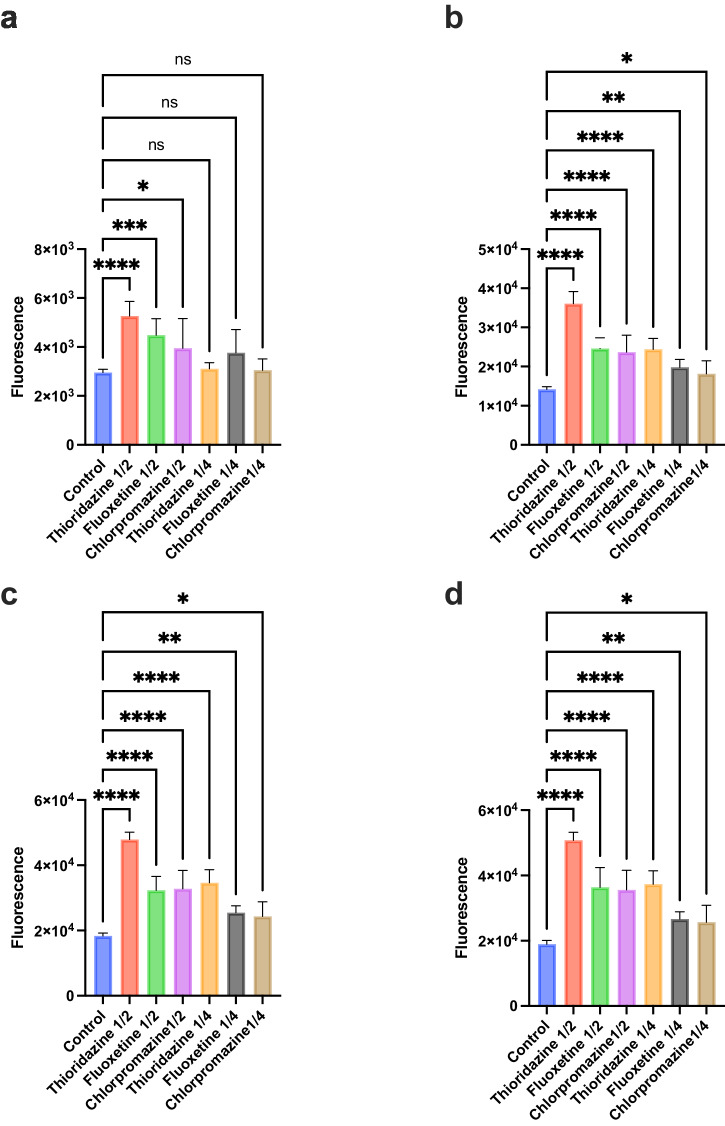
Increase in Hoechst fluorescence in *H. amylolyticum* CSM52 in the presence of ½ MIC and ¼ MIC of thioridazine, chlorpromazine and fluoxetine. Each time point (**a** 12 min, **b** 60 min, **c** 120 min, **d** 174 min) contrasts fluorescence in the presence and absence of EPIs. Error bars indicate the mean ± standard deviation determined from replicates (*n* = *8*). Asterisks denote significant differences between the relevant EPI compared to control, with **** *p* < 0.0001, *** *p* < 0.001, and ** *p* < 0.01.

*H. amylolyticum* CSM52 was examined for the presence of active efflux systems by exposure to ½ MIC (4, 8, and 16 μg/mL) and ¼ MIC (2, 4 and 8 μg/mL) of the three previously described efflux pump inhibitors (EPIs), thioridazine, chlorpromazine, and fluoxetine, respectively (Fig. 2)^4^. At both tested concentrations, the presence of the three EPIs resulted in efflux inhibition in *H. amylolyticum* as indicated by the higher fluorescence due to Hoechst accumulation in the treated cells compared to the control (absence of EPIs). This increase in fluorescence was significant after both 12 and 60 min of exposure to ½ MIC and ¼ MIC of the three EPIs, respectively (Figs. 3a and 3b, respectively). A dose dependent effect is also observed for the three EPIs, where the level of Hoechst dye accumulation increases with increasing the EPI concentration. However, *H. amylolyticum* CSM52 exhibited greater inhibition by thioridazine, compared to fluoxetine and chlorpromazine, as indicated by the greater fluorescence accumulation in the presence of thioridazine at both concentrations.

### Antimicrobial susceptibility of *H. amylolyticum* CSM52 in the presence of EPIs

To determine if exposure of *H. amylolyticum* CSM52 to EPIs would increase its sensitivity to antimicrobial agents, MIC values were compared for a range of antimicrobials, in the presence and absence of EPIs. Generally, *H. amylolyticum* CSM52 exhibited inherent resistance/tolerance to a wide range of antimicrobials, including tetracycline, chloramphenicol, vancomycin and members of the aminoglycosides and fluroquinolones. However, significant decreases in MIC for several antimicrobials in the presence of EPIs were observed, including tetracycline, novobiocin, erythromycin, bacitracin, rifampicin, and chloramphenicol as listed in Table 1.

**Table 1:** Influence of EPIs (¼ MIC) on the susceptibility of *H. amylolyticum* CSM52 to various antibiotics

Antimicrobial	Control	Fluoxetine	Thioridazine	Chlorpromazine
Tetracycline	312	78	78	78
Novobiocin	0.06	≤0.002	0.008	≤0.002
Erythromycin	62.5	15.6	7.8	31.25
Rifampicin	15.6	7.8	3.9	7.8
Bacitracin	156	39	39	78
Chloramphenicol	250	62.5	62.5	62.5
Ciprofloxacin	23	23	23	23
Vancomycin	512	512	512	512
Norfloxacin	78	78	78	78
Gentamicin	1024	512	512	512
Neomycin	2500	2500	2500	2500

All concentrations are in µg/mL and were tested against a population density of 5 × 10^5^ CFU/mL of *H. amylolyticum* CSM52. (*n* = 8).

Fluoxetine was found to increase the sensitivity of *H. amylolyticum* to tetracycline, erythromycin, bacitracin, and chloramphenicol by fourfold, and to novobiocin by over 30-fold, despite *H. amylolyticum’s* inherently high sensitivity to novobiocin (MIC value of 0.06 µg/mL). Thioridazine exhibited a similar effect on tetracycline, rifampicin, bacitracin, and chloramphenicol susceptibility, however, uniquely it increased the erythromycin sensitivity eightfold and novobiocin by eightfold. Chlorpromazine, in contrast, selectively enhanced sensitivity only to tetracycline, chloramphenicol, and novobiocin (over 30-fold). Interestingly, thioridazine consistently resulted in equivalent or greater increases in susceptibility compared to the other EPIs. These susceptibility shifts are in agreement with the Hoechst dye accumulation assays, where thioridazine also resulted in the most significant efflux inhibition. These data confirm thioridazine as a potent inhibitor of *H. amylolyticum* antibiotic efflux systems.

### Hoechst dye accumulation in *E. coli* clones harbouring putative MATE genes

**Fig. 4:**
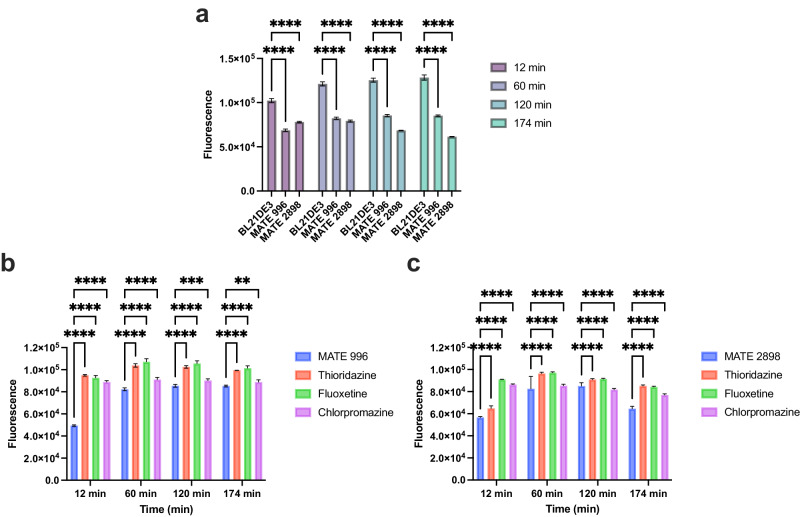
Quantification of Hoechst dye efflux from *E*. *coli* clones harbouring MATE-996 or MATE-2898 efflux genes. Variation in Hoechst dye accumulation in *E. coli* clones harbouring either MATE-996 or MATE-2898 genes compared to *E. coli* BL21DE3 (control) (**a**). The effect of EPIs on Hoechst dye accumulation in *E. coli* cells expressing MATE-996, treated with thioridazine, fluoxetine, and chlorpromazine at 12, 60, 120, and 174 min (**b**). Hoechst dye accumulation in *E. coli* cells expressing MATE-2898, treated with thioridazine, fluoxetine, and chlorpromazine at 12, 60, 120, and 174 min (**c**). *(n* = *3)*. Asterisks indicate statistical significance compared to the control cells, with **** *p* < 0.0001, *** *p* < 0.001, and ** *p* < 0.01.

To further confirm the functionality of the identified MATE pumps (MATE-996 and MATE-2898), the corresponding genes were cloned into *E. coli* BL21DE3, in a similar approach adopted previously for other efflux pumps^22,23^. This strategy was necessary because of the recognised challenges in the direct analysis of archaeal genomes and their expression systems, which are often compounded by inaccuracies in genome annotation^24,25^.The resultant clones exhibited reduced Hoechst dye accumulation compared to the untransformed *E. coli* at all measured times (Fig. 4a), confirming these genes role in functional efflux pump expression. Additionally, *E. coli* clones expressing MATE-996 (Fig. 4b) and MATE-2898 (Fig. 4c) exhibited a marked increase in fluorescence upon treatment with thioridazine, fluoxetine, and chlorpromazine at all time points compared to the control (*p* < 0.0001). These results provide strong evidence of the role of MATE-2898 and MATE-996 proteins in mediating Hoechst dye efflux and highlight the effectiveness of thioridazine, fluoxetine, and chlorpromazine as inhibitors of archaeal MATE pump activity in an *E. coli* model system, thereby offering an indirect yet valuable insights into the potential functionality of these efflux pumps in their native archaeal context.

### Interaction of antibiotics and EPIs with *H. amylolyticum* CSM52 MATE transporters

**Fig. 5:**
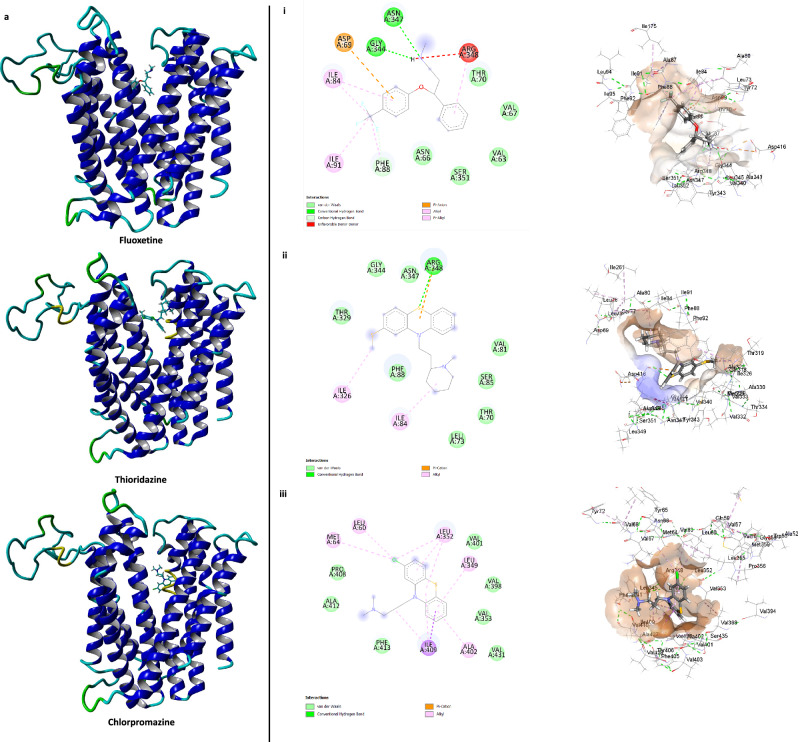

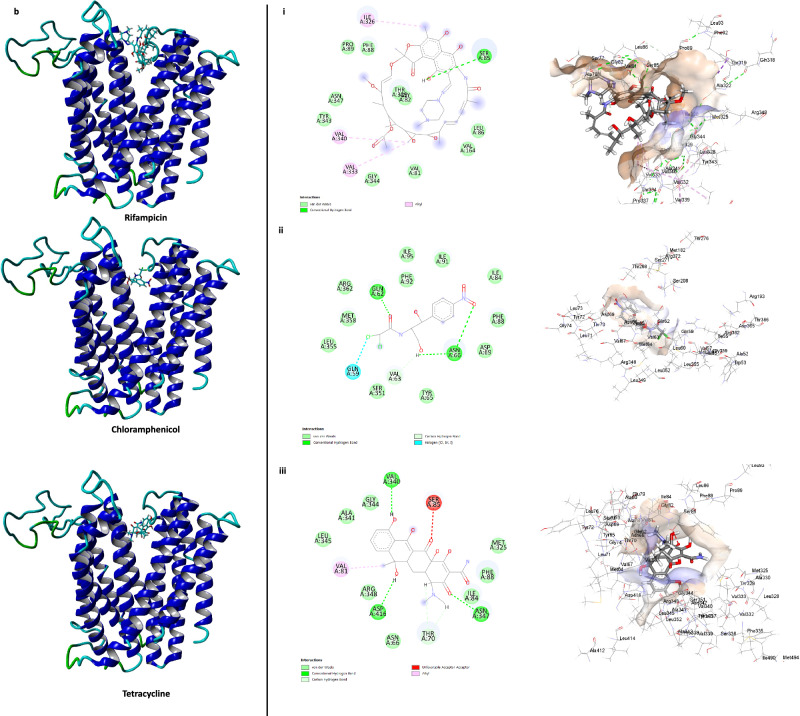
Interactions of the EPIs, fluoxetine, thioridazine and chlorpromazine and the substrate antibiotics rifampicin, chloramphenicol, and tetracycline within the channel region of MATE996 transporter. 3D structure of MATE996 with EPIs (**a**) and substrate antibiotics (**b**). Binding sites and key interactions between MATE996 and EPIs (**a i**–**iii**) and substrate antibiotics (**b i–iii**).

Molecular modelling which aimed to investigate the interactions between the two MATE transporters, the EPIs, and the selected antibiotics (tetracycline, chloramphenicol, and rifampicin) which exhibited potentiation in their presence, was conducted using YASARA Structure software Version 21.6.17. Ciprofloxacin, unaffected by the presence of EPIs, served as a control. In the case of the MATE-996 transporter, all three EPIs were found to bind to the channel region (Fig. 5a). Both fluoxetine and thioridazine either directly interacted with, or in proximity with, Thr70, Ile84, Phe88, Gly344, Asn347 and Arg348 within the binding pocket (Fig. 5a i and ii) suggesting they are occupying the same binding pocket. Chlorpromazine occupied an adjacent binding pocket, potentially blocking antibiotic transport via steric hindrance within the channel (Fig. 5a iii). Interestingly, all three substrate antibiotics interacted within a similar region of the channel (Fig. 5b i, ii, and iii), suggesting steric interactions with the EPIs that potentially inhibit efflux. However, ciprofloxacin interacted with a different binding pocket adjacent to the channel, explaining the EPIs’ inability to enhance its activity (Supplementary Fig. 1).

**Fig. 6:**
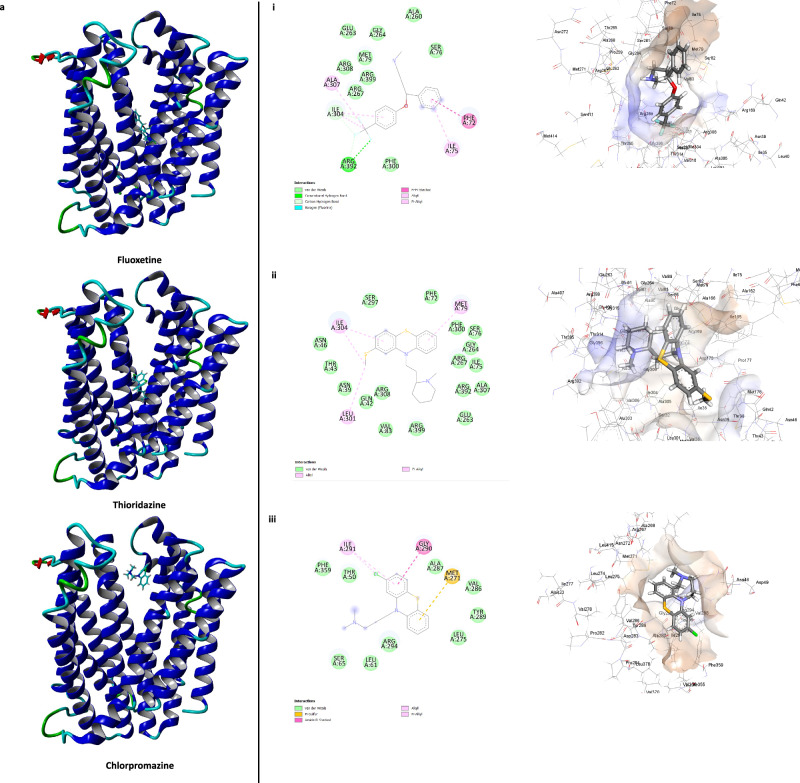

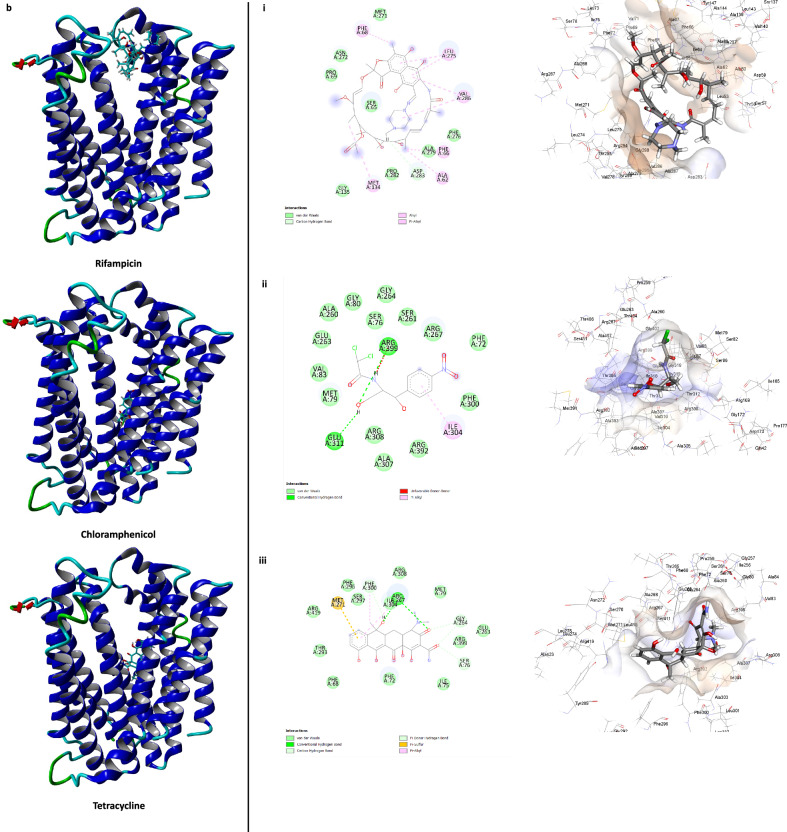
Interactions of the EPIs, fluoxetine, thioridazine and chlorpromazine and the substrate antibiotics rifampicin, chloramphenicol, and tetracycline within the channel region of MATE2898 transporter. 3D structure of MATE-2898 with EPIs (**a**) and substrate antibiotics (**b**). Binding sites and key interactions between MATE-2898 and EPIs (**a i–iii**) and substrate antibiotics (**b i**–**iii**).

In the case of the MATE2898 transporter, all three EPIs occupied the channel region. Thioridazine and fluoxetine interacted within the same binding pocket inside the deep channel architecture, while chlorpromazine interacted near the transporter’s opening (Fig. 6a). Thioridazine and fluoxetine either directly interacted with, or in proximity with, Phe72, Ile75, Ser76, Met79, Phe300, Ile304 and Arg308 within the binding site (Fig. 6a i, ii and iii). Interestingly, the substrate antibiotics chloramphenicol and tetracycline interacted with the same pocket as fluoxetine and thioridazine (Fig. 6b ii and iii), while rifampicin interacted near the channel’s opening. It is important to note that the larger molecular size of rifampicin may affect its interaction with the deep channel architecture due to steric considerations in the modelling software, YASARA structure. However, in the biological system, the presence of EPIs is likely to affect its transport within these pumps due to their occupancy of the channel.

### Evolutionary conservation and phylogenetic analysis of the *H. amylolyticum* MATE efflux systems

**Fig. 7:**
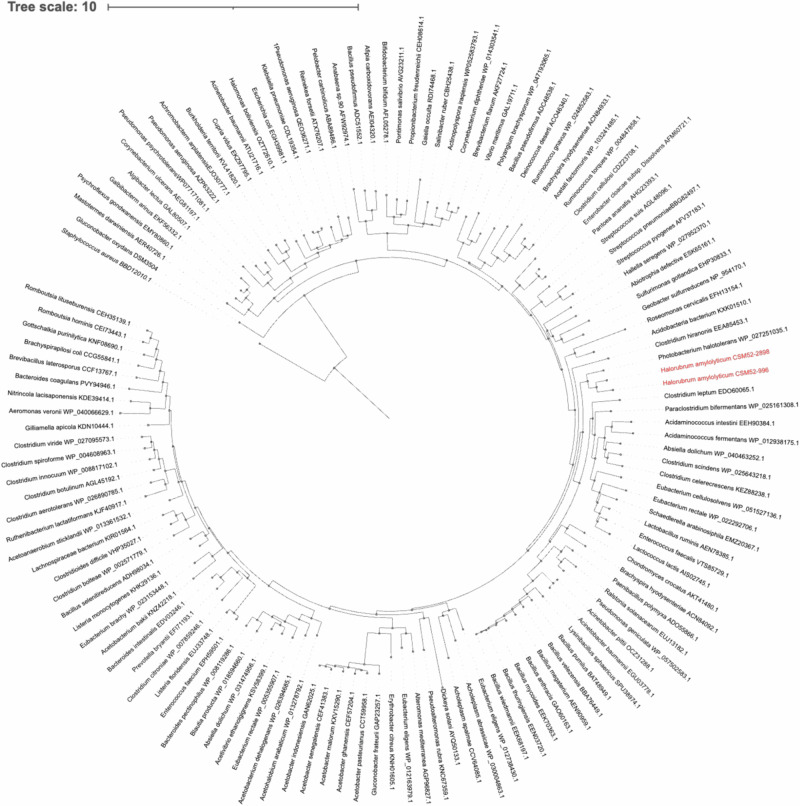
Molecular phylogenetic analysis based on the amino acid sequences of the MATE proteins from of *H. amylolyticum* CSM52 (in red) in comparison with MATE protein sequence from various bacterial species. The evolutionary history was inferred by using the Maximum Likelihood method based on the JTT matrix-based model. The tree with the highest log likelihood (−49977.93) is shown. The tree is drawn to scale, with branch lengths measured in the number of substitutions per site. The analysis involved 83 amino acid sequences. There was a total of 310 positions in the final dataset. Evolutionary analyses were conducted in FastTree (v5).

To elucidate the evolutionary relationships of the MATE efflux systems in *H. amylolyticum* CSM52, we conducted a phylogenetic analysis comparing the amino acid sequences of its putative MATE genes with those from a variety of bacterial species. These bacterial sequences were from representative, well-characterised MATE proteins that have been experimentally validated. The results, illustrated in Fig. 7, reveal a relatively low similarity between the sequences from *H. amylolyticum* CSM52 and those from the bacterial domain, underscoring the unique evolutionary trajectory of these archaeal MATE pumps. Both sequences were most similar to the halotolerant bacterium *Photobacterium halotoleran*s with a distance of 1.4 mutations/site.

## Discussion

Inherent antimicrobial resistance in archaea, evidenced by widespread resistance to antibiotics such as fosfomycin, vancomycin, and norfloxacin among others, has been well documented in the literature^12,26–32^. The broad-spectrum resistance of archaea is largely attributed to the differences in the outer envelope, including the absence of peptidoglycan in their cell walls, rendering them innately resistant to agents targeting peptidoglycan biosynthesis and cross-linking^32^. The antimicrobial susceptibility results in this study, reveal extensive intrinsic resistance to a spectrum of antimicrobials, including glycopeptides, aminoglycosides, macrolides, fluoroquinolones, tetracyclines, and chloramphenicol.

The increased susceptibility of *H. amylolyticum* CSM52 to several antibiotics when exposed to known bacterial multidrug efflux transporter inhibitors (such as thioridazine as described in *Proteus mirabilis*^4^ and chlorpromazine in *Staphylococcus aureus*^33^) suggests mechanisms of resistance that could involve active efflux systems. Intrinsic resistance mechanisms, including the role of efflux pumps in antimicrobial resistance, remain largely unexplored in archaea, compared to bacterial systems^34–41^.

This observation, together with the increased accumulation of Hoechst dye in *H. amylolyticum* in the presence of these inhibitors, indicates the important functional role of efflux pumps in *H. amylolyticum*. Importantly with Hoechst 33342, following accumulation in MATE efflux pumps such as with LmrP^42^, the Hoechst substrate undergoes unique binding interactions that prevent it from being extruded from MATE pumps, unlike other multidrug transporters where it is not accumulated, suggesting a specificity of this dye for MATE pumps^43^.

To further investigate the role of MATE pumps, genomic analysis of *H. amylolyticum* CSM52 was conducted, and it revealed a diverse array of transporters, predominantly ABC transporters, which facilitate the efflux of a wide range of substrates across the cellular membrane. Of particular interest, were peg.996 and peg.2898, identified as part of the MATE family of MDR efflux pumps. While previous work has detailed the role of bacterial ABC transporters in resistance^44^, their presence or inhibition does not alter does not alter Hoechst dye accumulation as observed previously^45^. This further supports the inference that the observed dye accumulation is attributable to MATE transporter activity.

The use of the heterologous expression in *E. coli* to infer the functionality of MATE pumps in *H. amylolyticum* CSM52 has its limitations. However, it provided useful insights that demonstrated phenotypic parallels between the *E. coli* MATE-996 and MATE-2898 clones and the native pumps, particularly in response to EPIs. The results provided a persuasive argument for their conserved efflux capabilities, and these findings represent an integral step towards a more comprehensive understanding of MATE pumps in archaeal species. Additionally, this experiment relies on the specificity of EPIs known to target MATE transporters^46^, the consequent alteration in Hoechst dye accumulation, and the conservation of MATE genes themselves^47^ to provide indirect evidence for the activity of these pumps.

Therefore, to further characterise these MATE pumps their amino acid sequences were aligned to each other and against a diverse selection of bacterial representatives. The phylogenetic analysis revealed a low similarity between the MATE pump sequences and representative genes from the bacterial domain. Interestingly, both sequences displayed the greatest resemblance to the halotolerant bacterium *Photobacterium halotolerans*, with a high mutation rate of 1.4. This result suggests that the MATE pumps of *H. amylolyticum* CSM52 diverged significantly from their bacterial counterparts, hinting at a unique evolutionary lineage of MATE efflux pumps in this archaeon. Interestingly, while the WGS annotations of *H. amylolyticum* CSM52 showed a closer resemblance to plant MATE transporters, bacterial MATE inhibitors still exhibited inhibitory effects on this archaeon. This observation is in agreement with findings in *Pyrococcus furiosus*, where a MATE transporter was shown to be inhibited by CCCP, and norfloxacin was identified as a substrate of the *P. furiosus* MATE transporter^8^. Furthermore, previous studies have identified amino acids in MATE transporters, which aid in correlating structure-function relationships and could be similar in archaeal MATE transporters^48^.

The structural basis and mechanisms for drug extrusion by MATE transporters, their conserved nature across domains, as well as their contribution to multidrug resistance, have been well established and provide a functional parallel to our findings in archaea^8^. The molecular modelling has provided insights into the intricate, putative interactions between EPIs and MATE efflux pumps in *H. amylolyticum* CSM52. The results demonstrate that EPIs and substrate antibiotics share a preference for similar binding pockets within the transporter’s channel architecture, a discovery that is consistent with the action of MATE transporters as documented in other organisms. For instance, structural analyses of bacterial MATE transporters have revealed H-bonding networks and multidrug-binding sites supporting a direct-competition mechanism for EPIs^49^. Additionally, our computational predictions for the binding behaviour of EPIs in *H. amylolyticum* CSM52 resonate with findings from the MATE transporters of other species, such as NorM-NG, where inhibitors like verapamil bind within the multidrug-binding site adjacent to the membrane-periplasm interface^50^. This corroborates our hypothesis of a conserved mechanism of action across MATE efflux pumps and reinforces the validity of our modelling results^51^. In contrast, ciprofloxacin, a non-substrate antibiotic, showed an affinity for a different pocket, suggesting a specific steric hindrance mechanism at play. Such specificity in interaction points to the nuanced nature of substrate and inhibitor binding within MATE pumps, where EPI occupancy could impede the dynamics of antibiotic efflux, thereby increasing the intracellular antibiotic concentration and potentiating their efficacy.

Our comprehensive genomic and phylogenetic analysis, inhibitor-based functional assays, and molecular modelling, collectively indicate the involvement of functional MATE efflux pumps. The results further demonstrate the involvement of MATE efflux pumps through the observed increased antibiotic susceptibility of *H. amylolyticum* CSM52 in the presence of EPIs. Indeed, while our data are strongly suggestive of the role that MATE efflux pumps are the drivers of dye and antibiotic efflux, without direct proteomic evidence or RT-PCR validation, which are challenging due to the organism’s slow growth and high salt requirements, the contribution of other transporters cannot be completely dismissed. As noted by Schulze et al., isolation and identification of small proteins and integral membrane proteins present considerable difficulties^52^. Haloarchaea proteomes are intricately adapted to high saline conditions, significantly impacting protein structure and solubility^53^. Given these considerations, standard approaches such as protein extraction and mass spectrometry require substantial adaptation, necessitating advanced, high-resolution instrumentation and specialised bioinformatics tools. Despite these limitations, our findings are substantiated by robust genomic data and functional assays, and we commit to further validating these results with protein-level evidence in future work.

Whilst the precise physiological functions of MATE efflux pumps, and indeed efflux mechanisms in general, in archaea, are largely unknown, bacterial multidrug efflux systems have been shown to play important roles in a wide range of processes, not just antimicrobial resistance^54^. The insights from this study may reveal crucial mechanisms by which archaea, thriving in complex environmental microbiota, adapt to antimicrobial challenges and may potentially harbour and disseminate antimicrobial resistance genes.

## Methods

### Strain isolation, sequencing, and annotation

*H. amylolyticum* CSM52 was obtained from Kilroot Salt Mine, located in Carrickfergus, County Antrim, Northern Ireland. The strain was isolated from a saturated brine pool and has been characterised and identified as a haloarchaea species using both 16 S rRNA sequencing using the Archaeal 16 S primers (Arch21F and Arch958R) and WGS^18^. The genome was annotated by uploading into the RAST server (rast.nmpdr.org), a fully automated service for annotating bacterial and archaeal genomes, and uses subsystem-based assertions to identify and assign functions to protein-encoding genes^21^. The nucleotide sequences of the genome was deposited as a fasta file at Zenodo (10.5281/zenodo.12626991).

### Reagents used in this study

Rifampicin, bacitracin, erythromycin, neomycin, tetracycline, novobiocin, vancomycin, thioridazine HCl, chlorpromazine HCl and gentamicin were obtained from Sigma Aldrich (UK). Fluoxetine and chloramphenicol were purchased from Fluorochem (UK). Norfloxacin was purchased from Alfa Aesar (UK). Ciprofloxacin was purchased from Fluka (UK), and Hoechst H33342 dye was obtained from Cayman Chemical (UK).

### Antimicrobial susceptibility testing

The MICs of the EPIs thioridazine, chlorpromazine and fluoxetine against *H. amylolyticum* CSM52 were determined in Payne’s medium according to NCCLS guidelines^55^ with slight modifications. The minimum inhibitory concentration (MIC) of each antimicrobial was then determined in the presence of sub-MIC values of each inhibitor (with a final concentration of ¼ MIC of the EPIs)^41,56^.

### Hoechst dye accumulation in *H. amylolyticum* CSM52

To determine the presence of functional efflux mechanisms in *H. amylolyticum* CSM52 (as inferred from the WGS and annotation), a standard Hoechst 33342 intracellular dye accumulation assay was performed in *H. amylolyticum* CSM52 over a 3-h incubation period at 37 °C. The Hoechst accumulation assay was performed according to the previously published method of Coldham et al.^57^. Essentially, *H. amylolyticum* CSM52 was grown to mid-exponential phase, cells were then pelleted and washed three times with phosphate buffer saline containing 20% NaCl (PB20%), then resuspended in PB20% to an optical density at 580 nm (OD_580_) of 0.2. Then 178 µL of cell suspension was mixed with 2 µL of EPI stock solution in black-clear bottom 96-well plate (Corning®), to a final concentration of ¼ MIC or ½ MIC of EPIs. Next, H33342 dye (20 µL) was added to a final concentration of 25 µM. Controls were included by the addition of PB20% instead of the corresponding EPI. A heat-inactivated sample (178 µl, incubated at 99 °C for 40 min in a thermocycler) with PB20%, instead of the corresponding EPI, was also included in the test as a positive control. Each test parameter was run in eight wells. The plate was then directly transferred to the plate reader to measure the fluorescence inside the wells. Fluorescence was measured using FLUOstar Omega (Ortenberg, Germany) microplate reader over a 3 h incubation period at 37 °C. Measurements were taken from the top of the wells using excitation and emission filters of 355 and 460 nm with 5 flashes/well, respectively. Readings were taken for 30 cycles with a 75 s delay between cycles and a gain multiplier of 1460.

### MATE gene cloning in *E. coli* BL21DE3 and culturing

The gene construct containing putative MATE genes was synthesised by GenScript (Netherlands). Synthesised pET28a (+) plasmid containing each gene of the MATE efflux pumps was cloned using BamHI and HindIII restriction sites, which were used to transform BL21DE3 cells. The transformation of BL21DE3 was also performed by GenScript (Netherlands).The two *E. coli* clones and the control (untransformed *E. coli* BL21DE3) were cultured from frozen stock in lysogeny broth (LB) medium containing 50 µg/mL kanamycin and incubated at 37 °C overnight. 1 mL of each culture was re-inoculated in 100 mL kanamycin-supplemented LB medium to mid-exponential phase (OD_580_ of 0.4–0.6). Gene expression was induced with 0.8 mM Isopropyl β-d-1-thiogalactopyranoside at room temperature overnight. To determine whether functional MATE efflux pumps were successfully expressed in *E. coli* clones, Hoechst 33342 accumulation in each of the three clones was measured over a 3-h incubation time, as described previously. Additionally, this experiment was repeated in the presence of the EPIs fluoxetine, thioridazine and chlorpromazine to determine the impact of inhibitor on Hoechst accumulation and, therefore, MATE function.

### Molecular docking of EPIs and antibiotics with *H. amylolyticum* CSM52 MATE transporters

Homology models of both MATE996 and MATE2898 were initially developed using YASARA Structure software Version 21.6.17 with PDB ID 3WBN as the template. The models were validated using Alphafold2. Energy minimisation and a 50 ns molecular dynamics simulation were performed with YASARA Structure using the AMBER16 force field. A blind global molecular docking study was conducted using YASARA Structure’s dock.run.mcr macro, which employs the AutoDock Vina scoring function by default, for finding the best binding site of all EPISs and substrate antibiotics by exploring all probable binding cavities of the proteins. YASARA Structure utilises shape complementarity, energy minimisation, and scoring functions to predict ligand binding modes and affinity. Finally, 2D ligand–protein interaction maps, 3D binding pocket of EPIS and substrate antibiotics, and the Figures showing channel architecture were generated using BIOVIA Discovery Studio Visualizer 2021.

### Phylogenetic analysis of the *H. amylolyticum* MATE efflux systems

Bacterial sequences of the MATE efflux pump were obtained from the NCBI GenBank database server (https://www.ncbi.nlm.nih.gov/protein). A phylogenetic tree was constructed based on the alignment of the amino acid sequences using CLUSTALw and MUSCLE. Evolutionary analyses were conducted in FastTree 2 with 1000 bootstrap replicates^58^, and visualised using the Interactive Tree Of Life (v5)^59^. The evolutionary relationships of both trees were inferred using the maximum likelihood method based on Jones–Taylor Thornton matrix model. Nearest*-*neighbour*-*interchange was used as a heuristic method to improve the likelihood of the tree^60^.

### Statistical analysis

Data analysis was performed using the statistics software GraphPad PRISM v9.4.5. All data are presented as means ± standard deviation unless otherwise stated. Statistical analyses were performed using data from three or more biological replicates, using ANOVA with Dunnett’s test.

## Supplementary information

Supplementary Information

## Data Availability

The raw Illumina reads for CSM52 were deposited in the Sequence Read Archive (SRA) under accession number SRR28789096.

## References

[1] Zgurskaya, H. I. & Nikaido, H. Multidrug resistance mechanisms: drug efflux across two membranes. *Mol. Microbiol.* **37**, 219–225 (2000). 10931319 10.1046/j.1365-2958.2000.01926.x

[2] Nies, D. H. Efflux-mediated heavy metal resistance in prokaryotes. *FEMS Microbiol. Rev.* **27**, 313–339 (2003). 12829273 10.1016/S0168-6445(03)00048-2

[3] Du, D. et al. Multidrug efflux pumps: structure, function and regulation. *Nat. Rev. Microbiol.* **16**, 523–539 (2018). 30002505 10.1038/s41579-018-0048-6

[4] Nzakizwanayo, J. et al. Fluoxetine and thioridazine inhibit efflux and attenuate crystalline biofilm formation by *Proteus mirabilis*. *Sci. Rep.* **7**, 1–14 (2017). 28939900 10.1038/s41598-017-12445-w PMC5610337

[5] Alav, I., Sutton, J. M. & Rahman, K. M. Role of bacterial efflux pumps in biofilm formation. *J. Antimicrob. Chemother.* **73**, 2003–2020 (2018). 29506149 10.1093/jac/dky042

[6] Bartolucci, S. et al. Responding to toxic compounds: a genomic and functional overview of Archaea. *Front. Biosci. Landmark* **18**, 165–189 (2013). 10.2741/4094 23276916

[7] Ninio, S. & Schuldiner, S. Characterization of an archaeal multidrug transporter with a unique amino acid composition. *J. Biol. Chem.* **278**, 12000–12005 (2003). 12551892 10.1074/jbc.M213119200

[8] Tanaka, Y. et al. Structural basis for the drug extrusion mechanism by a MATE multidrug transporter. *Nature* **496**, 247–251 (2013). 23535598 10.1038/nature12014

[9] Jagessar, K. L., Mchaourab, H. S. & Claxton, D. P. The N-terminal domain of an archaeal multidrug and toxin extrusion (MATE) transporter mediates proton coupling required for prokaryotic drug resistance. *J. Biol. Chem.* **294**, 12807–12814 (2019). 31289123 10.1074/jbc.RA119.009195 PMC6709631

[10] Miyauchi, S., Komatsubara, M. & Kamo, N. In archaebacteria, there is a doxorubicin efflux pump similar to mammalian P-glycoprotein. *Biochim. Biophys. Acta* **1110**, 144–150 (1992). 1356441 10.1016/0005-2736(92)90351-l

[11] DeLong, E. F. Everything in moderation: archaea as ‘non-extremophiles’. *Curr. Opin. Genet. Dev.* **8**, 649–654 (1998). 9914204 10.1016/s0959-437x(98)80032-4

[12] Dridi, B. et al. The antimicrobial resistance pattern of cultured human methanogens reflects the unique phylogenetic position of archaea. *J. Antimicrob. Chemother.* **66**, 2038–2044 (2011). 21680581 10.1093/jac/dkr251

[13] Lepp, P. W. et al. Methanogenic archaea and human periodontal disease. *Proc. Natl Acad. Sci. USA* **101**, 6176–6181 (2004). 15067114 10.1073/pnas.0308766101 PMC395942

[14] Drancourt, M. et al. Evidence of archaeal methanogens in brain abscess. *Clin. Infect. Dis.* **65**, 1–5 (2017). 28379309 10.1093/cid/cix286

[15] Wilmes, P. et al. *An Archaeal Compound as a Driver of Parkinson’s Disease Pathogenesis*. (2022).

[16] Yang, Y. et al. The fate of antibiotic resistance genes and their association with bacterial and archaeal communities during advanced treatment of pig farm wastewater. *Sci. Total Environ.* **851**, 158364 (2022). 36041618 10.1016/j.scitotenv.2022.158364

[17] Fuchsman, C. A. et al. Effect of the environment on horizontal gene transfer between bacteria and archaea. *PeerJ* **5**, e3865 (2017). 28975058 10.7717/peerj.3865 PMC5624296

[18] Thompson, T. P., Busetti, A. & Gilmore, B. F. Quorum sensing in *Halorubrum saccharovorum* facilitates cross-domain signaling between archaea and bacteria. *Microorganisms* **11**(5), 1271 (2023). 37317245 10.3390/microorganisms11051271 PMC10223598

[19] Megaw, J. et al. Profiling the microbial community of a *Triassic halite* deposit in Northern Ireland: an environment with significant potential for biodiscovery. *FEMS Microbiol. Lett.* **366**, fnz242 (2019). 31778179 10.1093/femsle/fnz242

[20] Thompson, T. P. et al. Microbiology of a NaCl stalactite ‘salticle’ in Triassic halite. *Environ. Microbiol.* **23**, 3881–3895 (2021). 33848049 10.1111/1462-2920.15524

[21] Aziz, R. K. et al. The RAST Server: rapid annotations using subsystems technology. *BMC Genomics* **9**, 1–15 (2008). 18261238 10.1186/1471-2164-9-75 PMC2265698

[22] Li, X.-Z. & Nikaido, H. Efflux-mediated drug resistance in bacteria: an update. *Drugs* **69**, 1555–1623 (2009). 19678712 10.2165/11317030-000000000-00000 PMC2847397

[23] Ogawa, W. et al. Characterization of MATE-type multidrug efflux pumps from *Klebsiella pneumoniae* MGH78578. *PloS ONE* **10**, e0121619 (2015). 25807080 10.1371/journal.pone.0121619 PMC4373734

[24] Overbeek, R. et al. Annotation of bacterial and archaeal genomes: improving accuracy and consistency. *Chem. Rev.* **107**, 3431–3447 (2007). 17658903 10.1021/cr068308h

[25] Brent, M. R. Steady progress and recent breakthroughs in the accuracy of automated genome annotation. *Nat. Rev. Genet.* **9**, 62–73 (2008). 18087260 10.1038/nrg2220

[26] Jones, J., Bowers, B. & Stadtman, T. C. *Methanococcus vannielii*: ultrastructure and sensitivity to detergents and antibiotics. *J. Bacteriol.* **130**, 1357–1363 (1977). 863858 10.1128/jb.130.3.1357-1363.1977 PMC235360

[27] Pecher, T. & Böck, A. In vivo susceptibility of halophilic and methanogenic organisms to protein synthesis inhibitors. *FEMS Microbiol. Lett.* **10**, 295–297 (1981).

[28] Miller, T. L. et al. Isolation of Methanobrevibacter smithii from human feces. *Appl. Environ. Microbiol.* **43**, 227–232 (1982). 6798932 10.1128/aem.43.1.227-232.1982 PMC241804

[29] Sioud, M. et al. Coumarin and quinolone action in archaebacteria: evidence for the presence of a DNA gyrase-like enzyme. *J. Bacteriol.* **170**, 946–953 (1988). 2828337 10.1128/jb.170.2.946-953.1988 PMC210746

[30] Kandler, O. & König, H. Cell wall polymers in Archaea (Archaebacteria). *Cell. Mol. Life Sci. CMLS* **54**, 305–308 (1998). 9614965 10.1007/s000180050156 PMC11147200

[31] Dermoumi, H. L. & Ansorg, R. A. Isolation and antimicrobial susceptibility testing of fecal strains of the archaeon *Methanobrevibacter smithii*. *Chemotherapy* **47**, 177–183 (2001). 11306786 10.1159/000063219

[32] Khelaifia, S. & Drancourt, M. Susceptibility of archaea to antimicrobial agents: applications to clinical microbiology. *Clin. Microbiol. Infect.* **18**(9), 841–848 (2012). 22748132 10.1111/j.1469-0691.2012.03913.x

[33] Kaatz, G. W. et al. Phenothiazines and thioxanthenes inhibit multidrug efflux pump activity in *Staphylococcus aureus*. *Antimicrob. Agents Chemother.* **47**, 719–726 (2003). 12543683 10.1128/AAC.47.2.719-726.2003 PMC151737

[34] Chen, J. et al. VmrA, a member of a novel class of Na+-coupled multidrug efflux pumps from *Vibrio parahaemolyticus*. *J. Bacteriol.* **184**, 572–576 (2002). 11751837 10.1128/JB.184.2.572-576.2002 PMC139572

[35] Jo, J. T., Brinkman, F. S. & Hancock, R. E. Aminoglycoside efflux in *Pseudomonas aeruginosa*: involvement of novel outer membrane proteins. *Antimicrob. Agents Chemother.* **47**, 1101–1111 (2003). 12604548 10.1128/AAC.47.3.1101-1111.2003 PMC149301

[36] Su, X.-Z. et al. AbeM, an H+-coupled *Acinetobacter baumannii* multidrug efflux pump belonging to the MATE family of transporters. *Antimicrob. agents Chemother.* **49**, 4362–4364 (2005). 16189122 10.1128/AAC.49.10.4362-4364.2005 PMC1251516

[37] Adabi, M. et al. Spread of efflux pump overexpressing-mediated fluoroquinolone resistance and multidrug resistance in *Pseudomonas aeruginosa* by using an efflux pump inhibitor. *Infect. Chemother.* **47**, 98 (2015). 26157587 10.3947/ic.2015.47.2.98 PMC4495281

[38] Zhu, M. & Dai, X. High salt cross-protects *Escherichia coli* from antibiotic treatment through increasing efflux pump expression. *Msphere* **3**, e00095–18 (2018). 29643076 10.1128/mSphere.00095-18 PMC5909119

[39] Umar, F. et al. Verapamil as an efflux inhibitor against drug resistant *Mycobacterium tuberculosis*: a review. *Syst. Rev. Pharm.* **10**, 22 (2019).

[40] Ebbensgaard, A. E., Løbner-Olesen, A. & Frimodt-Møller, J. The role of efflux pumps in the transition from low-level to clinical antibiotic resistance. *Antibiotics* **9**(12), 855 (2020). 33266054 10.3390/antibiotics9120855 PMC7760520

[41] Salah, A. N. et al. Cloning and sequencing of lsaE efflux pump gene from MDR Enterococci and its role in erythromycin resistance. *Infect. Genet. Evolut.* **94**, 105010 (2021). 10.1016/j.meegid.2021.105010 34293480

[42] Neuberger, A. & Van Veen, H. W. Hoechst 33342 is a hidden “Janus” amongst substrates for the multidrug efflux pump LmrP. *Plos ONE* **10**, e0141991 (2015). 26540112 10.1371/journal.pone.0141991 PMC4634932

[43] Swain, B. M. et al. Complexities of a protonatable substrate in measurements of Hoechst 33342 transport by multidrug transporter LmrP. *Sci. Rep.* **10**, 20026 (2020). 33208856 10.1038/s41598-020-76943-0 PMC7674423

[44] Dawson, R. J. & Locher, K. P. Structure of a bacterial multidrug ABC transporter. *Nature* **443**, 180–185 (2006). 16943773 10.1038/nature05155

[45] Murota, Y., Tabu, K. & Taga, T. Requirement of ABC transporter inhibition and Hoechst 33342 dye deprivation for the assessment of side population-defined C6 glioma stem cell metabolism using fluorescent probes. *BMC Cancer* **16**, 1–7 (2016). 10.1186/s12885-016-2895-8 PMC5097359 27814696

[46] Nzakizwanayo, J. et al. Fluoxetine and thioridazine inhibit efflux and attenuate crystalline biofilm formation by *Proteus mirabilis*. *Sci. Rep.* **7**, 12222 (2017). 28939900 10.1038/s41598-017-12445-w PMC5610337

[47] Brooks, L. E. et al. Quantifying the evolutionary conservation of genes encoding multidrug efflux pumps in the ESKAPE pathogens to identify antimicrobial drug targets. *Msystems* **3**, 00024–18 (2018). 10.1128/mSystems.00024-18 PMC5904435 29719870

[48] Otsuka, M. et al. Identification of essential amino acid residues of the NorM Na+/multidrug antiporter in *Vibrio parahaemolyticus*. *J. Bacteriol.* **187**, 1552–1558 (2005). 15716425 10.1128/JB.187.5.1552-1558.2005 PMC1064000

[49] Radchenko, M. et al. Structural basis for the blockade of MATE multidrug efflux pumps. *Nat. Commun.* **6**, 7995 (2015). 26246409 10.1038/ncomms8995 PMC4866600

[50] Kumawat, M. et al. Role of bacterial efflux pump proteins in antibiotic resistance across microbial species. *Microb. Pathogenesis* **181**, 106182 (2023). 10.1016/j.micpath.2023.106182 37263448

[51] Thakur, V., Uniyal, A. & Tiwari, V. A comprehensive review on pharmacology of efflux pumps and their inhibitors in antibiotic resistance. *Eur. J. Pharmacol.* **903**, 174151 (2021). 33964293 10.1016/j.ejphar.2021.174151

[52] Schulze, S. et al. The Archaeal Proteome Project advances knowledge about archaeal cell biology through comprehensive proteomics. *Nat. Commun.* **11**, 3145 (2020). 32561711 10.1038/s41467-020-16784-7 PMC7305310

[53] Carré, L. et al. Effects of chaotropic salts on global proteome stability in halophilic archaea: implications for life signatures on Mars. *Environ. Microbiol.* **25**, 2216–2230 (2023). 37349893 10.1111/1462-2920.16451

[54] Blanco, P. et al. Bacterial multidrug efflux pumps: much more than antibiotic resistance determinants. *Microorganisms* **4**, 14 (2016). 27681908 10.3390/microorganisms4010014 PMC5029519

[55] Matthew, A. et al. NCCLS, Methods for Dilution Antimicrobial Susceptibility Tests for Bacteria that Grow Aerobically; Approved Standard, M7-A7. (CLSI, Wayne, PA, 2006).

[56] Mombeshora, M. & Mukanganyama, S. Development of an accumulation assay and evaluation of the effects of efflux pump inhibitors on the retention of chlorhexidine digluconate in *Pseudomonas aeruginosa* and *Staphylococcus aureus*. *BMC Res. Notes* **10**, 1–9 (2017). 28747232 10.1186/s13104-017-2637-2 PMC5530522

[57] Coldham, N. G. et al. A 96-well plate fluorescence assay for assessment of cellular permeability and active efflux in *Salmonella enterica* serovar typhimurium and *Escherichia coli*. *J. Antimicrob. Chemother.* **65**, 1655–1663 (2010). 20513705 10.1093/jac/dkq169

[58] Price, M. N., Dehal, P. S. & Arkin, A. P. FastTree 2—approximately maximum-likelihood trees for large alignments. *PloS ONE* **5**, e9490 (2010). 20224823 10.1371/journal.pone.0009490 PMC2835736

[59] Letunic, I. & Bork, P. Interactive Tree Of Life (iTOL) v5: an online tool for phylogenetic tree display and annotation. *Nucleic Acids Res.* **49**, W293–W296 (2021). 33885785 10.1093/nar/gkab301 PMC8265157

[60] Jones, D. T., Taylor, W. R. & Thornton, J. M. The rapid generation of mutation data matrices from protein sequences. *Bioinformatics* **8**, 275–282 (1992). 10.1093/bioinformatics/8.3.275 1633570

