# Supplementary material for: Identification and characterisation of two functional antibiotic MATE efflux pumps in the archaeon Halorubrum amylolyticum
Source: NPJ Antimicrob Resist. 2024 Aug 2;2:21. doi: 10.1038/s44259-024-00036-5 (PMC11721430; doi:10.1038/s44259-024-00036-5)
Supplement: Supplementary file 1 — Supplementary Information [file 44259_2024_36_MOESM1_ESM.pdf]

Supplementary Information

**Supplementary Table 1: Genomic Features and Functional Categorization of *Halorubrum amylolyticum* CSM52**	
**Features**	**Role**
peg.3605	Ferric iron ABC transporter, ATP-binding protein
peg.2654	Ferric iron ABC transporter, iron-binding protein
peg.2659	Ferric iron ABC transporter, permease protein
peg.2890, peg.3037	Maltose/maltodextrin ABC transporter, MalE
peg.3511	Ribose ABC transport system, RbsC (TC 3.A.1.2.1)
peg.749, peg.3354, peg.3646	Glycerol-3-phosphate ABC transporter (TC 3.A.1.1.3)
peg.751, peg.1391, peg.1392, peg.3351	Glycerol-3-phosphate ABC transporter, UgpC (TC 3.A.1.1.3)
peg.3353	Glycerol-3-phosphate ABC transporter, UgpA (TC 3.A.1.1.3)
peg.3352	Glycerol-3-phosphate ABC transporter, UgpE (TC 3.A.1.1.3)
peg.2890, peg.3037	Maltose/maltodextrin ABC transporter, MalE
peg.748, peg.2891	Maltose/maltodextrin ABC transporter, MalF
peg.747, peg.2892, peg.3039	Maltose/maltodextrin ABC transporter, MalG
peg.2893	Multiple sugar ABC transporter, ATP-binding protein
peg.2809	Excinuclease ABC subunit A
peg.1239	Excinuclease ABC subunit C
peg.2857	Excinuclease ABC subunit B
peg.1436	ABC-type nitrate/sulfonate/bicarbonate transport system
peg.1419, peg.1861	Phosphate ABC transporter, PstS (TC 3.A.1.7.1)
peg.1419, peg.1861	Phosphate ABC transporter, PstS (TC 3.A.1.7.1)
peg.3240	ABC-type tungstate transport system, permease protein
peg.3241	ABC-type tungstate transport system, ATP-binding protein
peg.754, peg.3239	ABC-type tungstate transport system, periplasmic binding protein
peg.2540, peg.2738	Dipeptide-binding ABC transporter (TC 3.A.1.5.2)
peg.2170, peg.2484	Branched-chain amino acid ABC transporter (TC 3.A.1.4.1)
peg.2588	Phosphonate ABC transporter ATP-binding protein (TC 3.A.1.9.1)
peg.2587	Phosphonate ABC transporter (TC 3.A.1.9.1)
peg.2589	Phosphonate ABC transporter, phnE (TC 3.A.1.9.1)

peg.177, peg.2622, peg.2730	Oligopeptide ABC transporter, OppA (TC 3.A.1.5.1)
peg.72	Methionine ABC transporter ATP-binding protein
peg.1912	Urea ABC transporter, permease protein UrtB
peg.1913	Urea ABC transporter, substrate binding protein UrtA
peg.1910	Urea ABC transporter, ATPase protein UrtD
peg.1909	Urea ABC transporter, ATPase protein UrtE
peg.1911	Urea ABC transporter, permease protein UrtC
peg.2890, peg.3037	Maltose/maltodextrin ABC transporter, MalE
peg.2540, peg.2738	Dipeptide-binding ABC transporter (TC 3.A.1.5.2)
peg.1425, peg.3612	Vitamin B12 ABC transporter, B12-binding component BtuF
peg.1423	Vitamin B12 ABC transporter, ATPase component BtuD
peg.1424	Vitamin B12 ABC transporter, permease component BtuC
peg.996, peg.2898	MATE family of MDR efflux pumps
peg.2400	Cation efflux system protein CusA
peg.322	Probable Co/Zn/Cd efflux system membrane fusion protein
peg.1194, peg.1797	Magnesium and cobalt efflux protein CorC
peg.923, peg.1767	Arsenical pump-driving ATPase (EC 3.6.3.16)

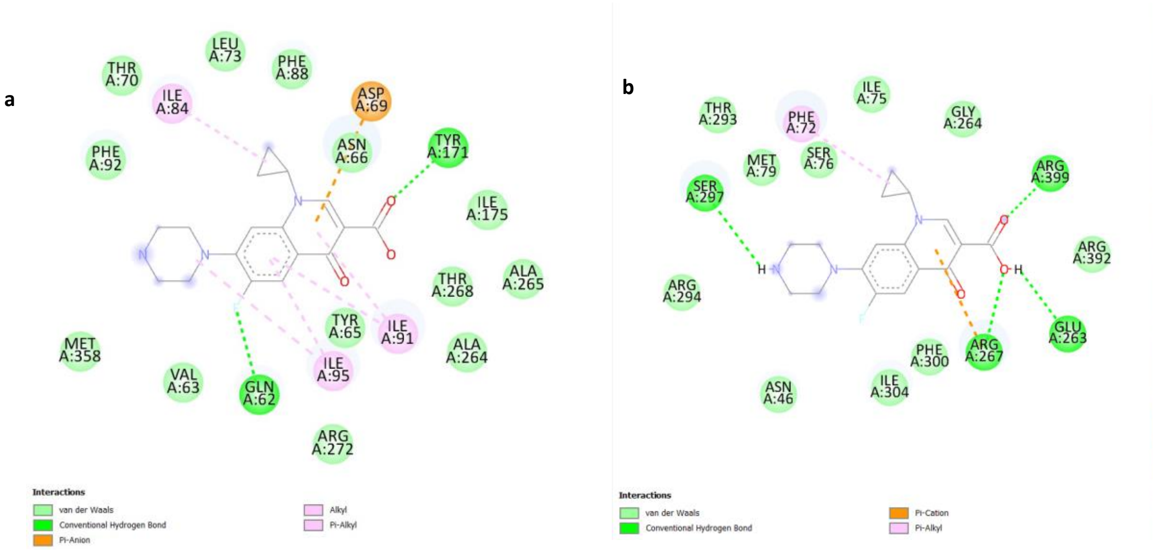

Supplementary FIG. 1 2D interactions map of ciprofloxacin with (**a**) MATE996 and (**b**) MATE2898 transporter.

12
13
